# Corticosteroid Receptor Modulation in the Dorsal Striatum Prevents Morphine‐Induced Place Preference and Reduction in Dopamine Transporter (DAT) Immunoreactivity in Rats

**DOI:** 10.1111/adb.70185

**Published:** 2026-09-13

**Authors:** Payman Raise‐Abdullahi, Fatemeh Rezamohammadi, Mehrnoush Rahmani, Abbas Ali Vafaei, Roghayeh Pakdel, Hamed Rashidipour, Ali Rashidy‐Pour

**Affiliations:** ^1^ Research Center of Physiology, Neuroscience Research Institute Semnan University of Medical Sciences Semnan Iran; ^2^ Department of Physiology, School of Medicine Semnan University of Medical Sciences Semnan Iran; ^3^ College of International Education Dalian Medical University Dalian China

**Keywords:** conditioned place preference, corticosteroid receptors, dorsal striatum, opioids, reward neurocircuitry, stress

## Abstract

Stress hormones and corticosteroid signalling influence dopaminergic transmission and reward‐related behaviours. The present study investigated whether pharmacological manipulation of corticosteroid signalling within the dorsal striatum influences morphine‐induced conditioned place preference (CPP) and dopamine transporter (DAT) regulation. Male Wistar rats were implanted with bilateral intra‐dorsal striatal cannulae and subjected to the CPP paradigm. Before conditioning sessions, animals received bilateral intra‐dorsal striatal infusions of corticosterone, the glucocorticoid receptor (GR) antagonist RU‐38486 or the mineralocorticoid receptor (MR) antagonist spironolactone. Behavioural preference, locomotor activity and DAT immunoreactivity were subsequently assessed. Morphine reliably induced CPP and reduced DAT immunoreactivity in the dorsal striatum. Corticosterone, RU‐38486 and spironolactone each significantly attenuated morphine‐induced CPP and prevented the reduction in DAT immunoreactivity without altering locomotor activity. The convergence of these pharmacologically distinct manipulations on similar behavioural and DAT outcomes suggests that perturbation of corticosteroid‐sensitive signalling within the dorsal striatum modulates opioid reward and associated dopaminergic plasticity. Importantly, the present findings do not establish whether corticosterone's effect is mediated by classical GR signalling, rapid corticosteroid actions or GR‐independent mechanisms. Given the role of the dorsal striatum in addiction‐related learning and the transition towards habitual and compulsive drug seeking, these findings identify corticosteroid–dopamine interactions within this region as an important mechanism for further investigation in opioid use disorders.

## Graphical abstract

Intra‐dorsal striatal corticosterone attenuated morphine‐induced CPP. GR and MR antagonists also attenuated morphine‐induced CPP. Morphine reduced DAT immunoreactivity, an effect prevented by CORT and antagonists. Distinct corticosteroid manipulations converged on similar reward and DAT outcomes. Dorsal striatal corticosteroid signalling is linked to morphine reward modulation.

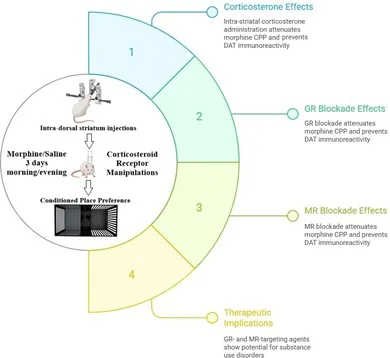

## 1 Introduction

Drug addiction arises from maladaptive neuroplasticity within reward circuits, where repeated drug exposure reshapes learning, memory and motivation [1]. Among drugs of abuse, opioids such as morphine robustly activate the mesolimbic dopamine system, producing reinforcement through dopamine release in the nucleus accumbens [2]. Beyond acute reward, chronic morphine exposure induces long‐lasting neuroadaptations across striatal regions, altering dendritic structure, synaptic plasticity and transmitter regulation [3]. These cellular changes are thought to underlie the transition from voluntary use to compulsive drug‐seeking behaviours.

Although the ventral striatum has classically been emphasized in opioid reward, emerging evidence highlights the dorsal striatum (DS) as a critical site in the consolidation of drug‐associated learning and habit formation [4, 5, 6]. The DS integrates motivational and sensorimotor inputs to support stimulus–response associations, and its dysfunction contributes to compulsive drug seeking [7, 8]. Chronic morphine exposure induces structural alterations in this region, including dendritic retraction and spine loss [3], underscoring its involvement in opioid‐related neuroplasticity. Dorsal striatal circuits integrate both reward and stress signals, influencing decision‐making, motivation and reinforcement learning [9]. Importantly, it is also sensitive to stress‐related modulation: Chronic opioids disrupt dorsal striatal plasticity [10], whereas stress signals acting on this region bias decision‐making and reinforcement learning [11]. Focusing on the DS, rather than the ventral striatum, allows us to investigate how stress–reward interactions contribute specifically to the habitual and compulsive aspects of opioid addiction, which remain less well defined.

The interplay between the brain's reward system and stress response mechanisms is central to understanding substance use disorders [12]. Glucocorticoid receptors (GRs) and mineralocorticoid receptors (MRs) are widely distributed throughout the brain [13] and play distinct roles in cognitive and affective regulation [14, 15, 16]. GRs are enriched in the hippocampus, prefrontal cortex and amygdala, where they contribute to memory consolidation, retrieval and extinction [13, 17]. MRs, also abundant in limbic regions [18], are more strongly associated with initial information encoding, including spatial and contextual learning [19]. Together, these receptors shape stress adaptation and learning, processes that are highly relevant to addiction. Their role in the striatum is of particular interest because corticosteroids modulate dopamine release, reuptake and receptor signalling [20]. For example, corticosterone influences striatal dopamine receptor activity [21], GRs regulate presynaptic dopamine release and transporter function [20], and corticosteroid signalling affects the expression of the dopamine transporter (DAT), a key determinant of extracellular dopamine [22]. Conversely, DAT activity can modulate corticosteroid receptor expression, indicating a bidirectional regulatory loop [23].

Corticosteroid receptors are well positioned to mediate this interaction. GRs and MRs are present across striatal subregions [24], where they regulate dopaminergic transmission and reward‐related behaviours [25]. Corticosterone modulates striatal dopamine receptor activity [21], whereas GRs influence dopamine release and reuptake [26]. Moreover, corticosteroids affect DAT expression, and the DAT itself may reciprocally modulate corticosteroid receptor signalling [23]. These bidirectional interactions suggest a potential mechanism through which stress hormones shape opioid reward.

Despite these insights, it remains unclear whether GRs and MRs in the DS differentially influence opioid reward and associated molecular adaptations. Previous studies have shown corticosteroid receptor involvement in psychostimulant responses [4, 5, 6], but their role in morphine‐induced conditioned place preference (CPP) and DAT regulation has not been directly tested. The CPP paradigm is one of the most widely established models for assessing the rewarding and motivational properties of drugs of abuse [27]. In this paradigm, animals form an association between a distinct environmental context and the pharmacological effects of a drug, and subsequent preference for the drug‐paired chamber is taken as an index of its reinforcing value. CPP is advantageous because it does not require operant training and directly integrates drug effects with contextual memory, thereby capturing both motivational and associative learning components of addiction. Its construct and predictive validity have been demonstrated across multiple drug classes, including opioids such as morphine, making it a standard preclinical approach for investigating the neurobiological substrates of drug reward and relapse vulnerability [28].

The present study addresses this gap by combining the CPP paradigm, bilateral intra‐DS pharmacological manipulations and immunohistochemistry. We hypothesized that GRs and MRs exert distinct yet convergent influences on morphine‐induced CPP and striatal DAT immunoreactivity. By delineating receptor‐specific contributions, this work aims to clarify corticosteroid–dopamine interactions in opioid addiction and highlight potential receptor targets for therapeutic intervention.

## 2 Material and Methods

### 2.1 Animals

In this study, 120 adult male Wistar rats weighing 200–250 g were utilized. They were provided unrestricted access to water and food and were housed in a controlled environment, where they experienced a consistent 12‐h light–dark cycle and a steady temperature of 22°C ± 2°C. Adherence to ethical guidelines outlined in the National Research Council's *Guide for the Care and Use of Laboratory Animals* was ensured throughout the experiments. Additionally, the study protocol was approved by the Research Ethics Committee of Semnan University of Medical Sciences, Semnan, Iran (IR.SEMUMS.REC.1398.260).

### 2.2 Drugs

Morphine sulfate (MOR) was obtained from Temad Co., Tehran, Iran. Corticosterone (CORT), a corticosteroid receptor ligand, RU38486 (RU), a GR blocker, and spironolactone (SP), an MR blocker, were sourced from Sigma Co., USA. MOR (10 mg/kg) was dissolved in sterile 0.9% saline (SAL) and administered 30 min before CPP conditioning via subcutaneous injection (s.c.) [27]. CORT (10 ng/0.3 μL), RU (10 or 100 ng/0.3 μL) and SP (10 or 100 ng/0.3 μL) were dissolved in 5% dimethyl sulfoxide (DMSO) and injected into the DS 30 min before CPP conditioning. Saline or DMSO (0.3 μL/side) was injected as the vehicle (VEH). The doses of MR and GR antagonists were chosen based on our pilot and previous studies [29]. Because RU, SP and CORT required dissolution in 5% DMSO, whereas morphine was dissolved in saline, we included both saline + intra‐DS DMSO and saline + intra‐DS saline vehicle groups. Statistical comparisons confirmed no significant differences in CPP scores or locomotor activity between these vehicle groups.

The doses used for intra‐dorsal striatal injections were selected based on prior intracerebral microinjection studies in striatal and limbic regions, as well as on pilot experiments. Intra‐striatal corticosterone was administered at 10 ng/0.3 μL per side, as previous dorsal striatal studies have shown that corticosterone produces dose‐dependent effects on striatal‐dependent learning and synaptic physiology in the low‐nanogram range (2–10 ng) and that 10 ng reliably modulates striatal function without producing gross motor confounds [30]. RU‐38486 was tested at 10 and 100 ng/0.3 μL per side (both doses reported in Section 3) based on published intracerebral GR blockade paradigms demonstrating the efficacy of low‐nanogram local administration at frontal/limbic and striatal sites. Spironolactone was examined at 10 and 100 ng/0.3 μL per side following prior central MR antagonist studies that used similar local doses and reported behavioural effects without systemic side effects [29]. Pilot dose‐finding studies in our laboratory confirmed that 10 ng/side corticosterone significantly modulated local striatal endpoints while preserving normal locomotor behaviour and that doses of 10–100 ng/side for antagonists produced detectable, dose‐dependent signal changes; full dose–response studies were beyond the scope of the current manuscript.

### 2.3 Experimental Groups

**FIGURE 1:**
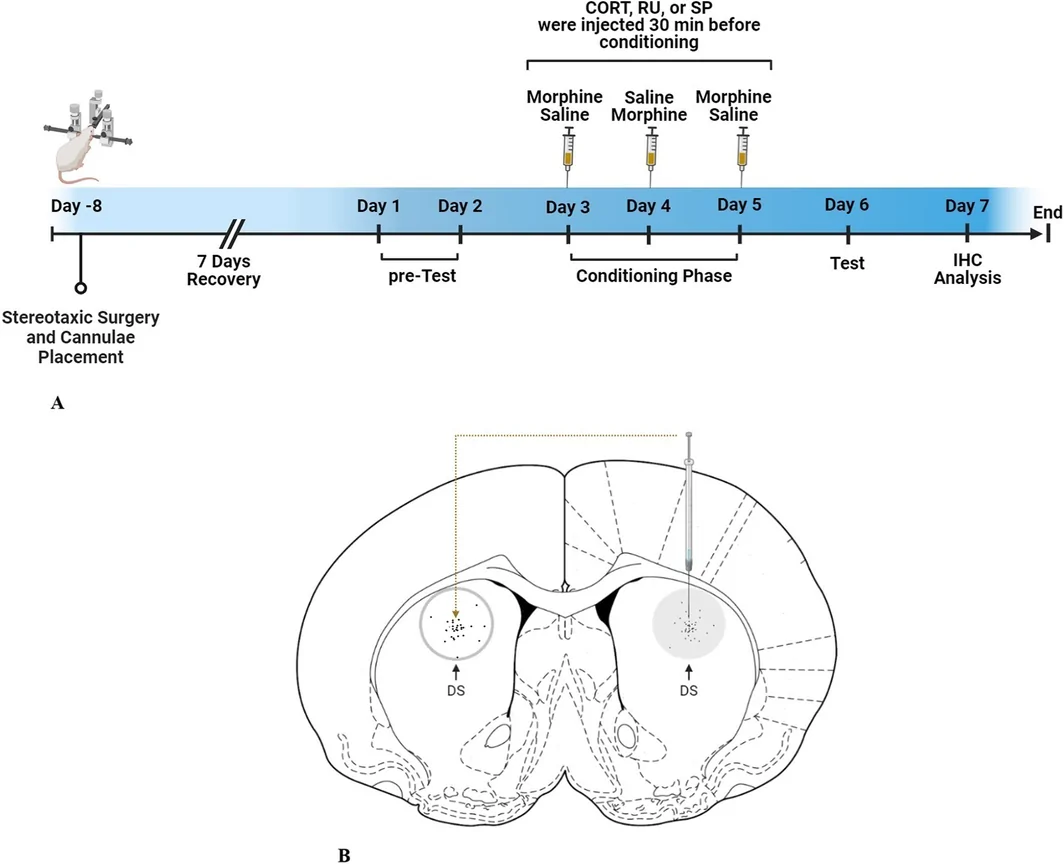
(A) Timeline of the experiment. (B) Schematic diagram illustrating bilateral intra‐dorsal striatum injection sites (both hemispheres). DS, dorsal striatum.

The timeline of the experiment is provided in Figure 1A. Rats were randomly divided into the following experimental groups (*n* = 10 in each group): (1) SAL‐VEH: received saline (2 mL/kg, s.c.) and intra‐DS vehicle (0.3 μL per side); (2) SAL‐CORT10: received saline and intra‐DS corticosterone (10 ng/0.3 μL per side); (3) MOR‐VEH: received morphine (10 mg/kg, s.c.) and intra‐DS vehicle; (4) MOR‐CORT10: received morphine and intra‐DS corticosterone (10 ng/0.3 μL per side); (5) SAL‐SP10: received saline and intra‐DS spironolactone (10 ng/0.3 μL per side); (6) MOR‐SP10: received morphine and intra‐DS spironolactone (10 ng/0.3 μL per side); (7) SAL‐SP100: received saline and intra‐DS spironolactone (100 ng/0.3 μL per side); (8) MOR‐SP100: received morphine and intra‐DS spironolactone (100 ng/0.3 μL per side); (9) SAL‐RU10: received saline and intra‐DS RU (10 ng/0.3 μL per side); (10) MOR‐RU10: received morphine and intra‐DS RU (10 ng/0.3 μL per side); (11) SAL‐RU100: received saline and intra‐DS RU (100 ng/0.3 μL per side); and (12) MOR‐RU100: received morphine and intra‐DS RU (100 ng/0.3 μL per side).

### 2.4 Stereotaxic Surgery and Drug Injection

Eight days before the experiment, rats were anaesthetized with intraperitoneal (i.p.) injections of ketamine (80 mg/kg) and xylazine (10 mg/kg). Animals were positioned in a stereotaxic apparatus, and the skull was levelled between bregma and lambda. Based on the Paxinos and Watson rat brain atlas [31], bilateral coordinates for the DS were identified (anteroposterior [AP]: +0.5 mm; mediolateral [ML]: ±3.0 mm; dorsoventral [DV]: −4.0 mm relative to bregma).

Small burr holes were drilled at the designated coordinates, and bilateral stainless steel guide cannulae (22 G) were implanted vertically above the DS in both hemispheres. Cannulae were secured to the skull using dental cement and anchoring screws. Following surgery, animals received ketoprofen (2.5 mg/kg, i.p.) for analgesia and penicillin G (3.5 mg/kg, i.m.) to prevent infection. Rats were allowed a minimum of 7 days for recovery prior to the start of behavioural procedures.

For intra‐dorsal striatal microinjections, internal injectors (28 ) extended 1 mm beyond the guide cannulae tips to reach the target DV coordinate (−4.0 mm). The injectors were connected via polyethylene tubing (PE‐20) to a 5‐μL Hamilton microsyringe. All injections were performed bilaterally, with a volume of 0.3 μL per hemisphere delivered over 60 s. Following infusion, the injectors were left in place for an additional 60 s to allow diffusion and minimize backflow along the cannula tract [32].

### 2.5 CPP

#### 2.5.1 CPP Apparatus

A three‐chamber CPP wooden box was utilized to evaluate morphine‐induced CPP [33]. The dimensions of the two primary chambers, A and B, were 30 × 30 × 40 cm. These chambers differed in their wall design: Both featured black walls with 2‐cm white stripes, but the stripes were vertical in Chamber A and horizontal in Chamber B. The third chamber, designated as C, was red and measured 30 × 15 × 40 cm, serving as a connector between Chambers A and B. Guillotine doors separated Chambers A and B from Chamber C. Upon removal of these doors, the animal could freely traverse Chambers A and B through the tunnel in Chamber C. A video camera positioned atop the CPP apparatus continuously monitored and recorded animals' behaviour, which was subsequently analysed using EthoVision Software Version 7 (Noldus Information Technology).

#### 2.5.2 Pre‐Test

Animals underwent two pre‐test sessions on consecutive days to (i) habituate them to the apparatus and attenuate novelty‐driven exploration and (ii) identify and exclude individuals with strong unconditioned side biases (an exclusion criterion of > 360 s spent in one chamber was applied). The second pre‐test (Day 2) was used as the baseline (‘pre‐test’) for subsequent statistical comparisons with the post‐conditioning test, consistent with standard CPP practice. On the first and second days, the guillotine doors were removed, allowing each animal to freely explore the apparatus by being placed in chamber C for a 10‐min acclimation period. A camera recorded the time each rat spent in Chambers A and B. Any rat that spent more than 360 s in either Chamber A or B was excluded from the study, as this indicated a pre‐existing preference. Therefore, only animals that showed no preference for either chamber during this initial phase were included in the study.

Rats that spent more than 360 s in either chamber during the second pre‐test were excluded from further analysis to avoid baseline chamber bias. In total, 9 rats (7.5% of 120) were excluded. Exclusions were distributed evenly (0–1 animal per group), ensuring that final group sizes remained balanced.

#### 2.5.3 Conditioning Phase

The conditioning phase of the CPP paradigm was conducted over 3 consecutive days (Days 3–5). During this phase, rats underwent daily sessions designed to create associations between the effects of morphine and specific chamber environments. Each day consisted of two conditioning sessions separated by a 5‐h interval. In each session, rats were administered either morphine or saline and then immediately confined to either Chamber A or B for 30 min. The treatment and chamber pairing were alternated across sessions, ensuring that if a rat received morphine in Chamber A during the first session, it would receive saline in Chamber B during the second session, and vice versa. To control for potential inherent chamber preferences, the association between morphine and chambers was counterbalanced across each experimental group. Half of the rats in each group received morphine in Chamber A, whereas the other half received it in Chamber B. The specific daily protocols were as follows:

Days 3 and 5: Rats were weighed and injected with morphine in the morning session and saline in the evening session. Day 4: The order was reversed, with saline administered in the morning and morphine in the evening to maintain consistent exposure to both treatments across the conditioning phase. By the conclusion of Day 5, each rat had experienced three pairings of morphine with one specific chamber and three pairings of saline with the other chamber. This procedure was designed to establish a learned association between the rewarding effects of morphine and the environmental cues of one chamber, whereas the other chamber became associated with the neutral effects of saline. This carefully structured protocol ensures that any subsequent preference for one chamber over the other can be attributed to the learned association with morphine's effects rather than to inherent chamber characteristics or uneven drug exposure.

#### 2.5.4 Test

The post‐conditioning test was conducted on the sixth day. The guillotine door was removed, allowing each rat to move freely in all chambers for 10 min. Throughout this period, the time spent in Chambers A and B, as well as the distance travelled, was recorded. Subsequently, CPP scores were calculated as the change in time spent in the morphine‐paired chamber between the test and pre‐test sessions using the following formula:

$$\begin{array}{c} \mathrm{CPP}\;\text{score}=\left(\text{test morphine}-\text{paired time}\right)\\ -\left(\mathrm{pre}-\text{test morphine}-\text{paired time}\right). \end{array}$$

This calculation reflects the increase (or decrease) in preference for the drug‐paired chamber relative to baseline. Saline sessions were included to equate handling and injection exposure across days; they functioned as pseudo‐conditioning sessions rather than as independent behavioural controls. Thus, group comparisons focused on changes in preference for the morphine‐paired chamber relative to baseline [27, 34].

Following the CPP test, animals were sacrificed, and brain tissue was collected for subsequent histological and immunohistochemical analyses.

### 2.6 Histological Analysis

After behavioural testing, rats were deeply anaesthetized with CO_2_ and decapitated [35]. Brains were rapidly extracted and immediately immersed in 10% neutral buffered formalin to preserve tissue integrity. After 48 h of fixation, brains were transferred to a 30% sucrose solution in phosphate‐buffered saline (PBS) for cryoprotection. Frozen brains were sectioned coronally at 40 μm using a cryostat maintained at −20°C. Sections containing the DS were mounted on gelatin‐coated slides and stained with 0.1% cresyl violet solution for 5 min, followed by graded ethanol dehydration (70%, 95% and 100%), clearing in xylene and coverslipping with DPX mountant. Cresyl violet–stained sections were examined under a light microscope (Olympus, Japan) by an experimenter who was blind to the treatment conditions. Cannula tip locations were mapped onto the Paxinos and Watson rat brain atlas [31]. Only animals with bilateral injector tips located within the DS were included in the analysis. Rats with misplaced cannulae were excluded (< 10% of animals). A schematic illustration of all confirmed injection sites is presented in Figure 1B.

### 2.7 Immunohistochemistry Assay

For DAT immunohistochemistry, brain tissues were collected from the same cohort of animals following completion of the CPP behavioural procedure. After decapitation, brains were immersion‐fixed in 10% neutral buffered formalin for 24–48 h. After fixation, the tissues were dehydrated through an ascending series of ethanol solutions, cleared in xylene and embedded in paraffin wax. Serial coronal sections (5 μm) containing the DS were cut using a rotary microtome and mounted on glass slides.

Sections were deparaffinized in xylene, rehydrated through a descending ethanol series and subjected to antigen retrieval in boiling citrate buffer (0.01 M, pH 6.0). Non‐specific binding was blocked with 10% normal donkey serum in PBS containing 0.1% Tween 20 and 3% Triton X‐100 for 1 h at room temperature. Slides were then incubated overnight at 4°C with rabbit anti‐DAT primary antibody (Biorbyt Ltd., UK; orb101657; polyclonal; RRID: AB_2895683; working dilution 1:500 in PBS; single lot used throughout). After PBS washes, sections were incubated for 90 min at 37°C in the dark with FITC‐conjugated goat anti‐rabbit IgG(H + L) secondary antibody (Biorbyt Ltd.; orb688925; polyclonal; RRID: AB_2896112; working dilution 1:150). Nuclear counterstaining was performed with DAPI (Sigma, D9542, 75 μg/mL, 15 min at 37°C). Sections were mounted with glycerol/PBS solution and coverslipped.

For quality control and validation, negative controls (primary antibody omitted) and positive control tissue known to express DAT were included in each staining run. The observed striatal DAT distribution was consistent with previously reported anatomical localization [36]. All immunohistochemistry experiments were conducted with a single lot of antibody to minimize variability. Image acquisition was performed with a fluorescence microscope (Olympus, Japan) at 200× magnification under standardized exposure settings. DAPI staining was used to visualize cell nuclei and confirm tissue integrity and anatomical localization within the DS. Merged images (DAT + DAPI) were generated for qualitative visualization only. Quantitative analysis of DAT immunoreactivity was performed using the DAT fluorescence channel alone, with measurements of immunoreactive area and optical density within defined regions of interest in ImageJ. All analyses were conducted by an experimenter blinded to the treatment conditions. This immersion‐fixation/paraffin protocol, combined with antigen retrieval and standardized imaging, has been reported to preserve striatal DAT immunoreactivity reliably [36, 37].

### 2.8 Statistical Analysis

In final sample sizes, although each group was initially assigned 10 rats, the effective n per analysis varied slightly due to (i) pre‐test exclusions for innate chamber bias (> 360 s criterion), (ii) histological exclusions for misplaced cannulae (< 10% of cases) and (iii) occasional tissue loss during immunohistochemistry processing. As a result, final group sizes ranged from 9 to 10 for behavioural analyses and 4 for DAT immunohistochemistry. Exact sample sizes for each analysis are reported in the figure legends.

Data were analysed using GraphPad Prism Version 9.0.0. One‐way ANOVA, two‐way ANOVA or two‐way repeated measures ANOVA was employed depending on the experimental design. Tukey's post hoc test was used for multiple comparisons when significant differences were found. Results are expressed as mean ± SEM, and a *p*‐value of less than 0.05 was considered statistically significant.

## 3 Results

### 3.1 Effects of Intra‐DS Injection of Corticosterone on the Expression of Morphine‐Induced CPP

Pre‐test analysis confirmed that baseline side preferences did not differ between groups. Second pre‐test, Day 2: no significant main effects of morphine (*F*_1,33_ = 0.006, *p* = 0.93) or CORT (*F*_1,33_ = 0.01, *p* = 0.89) or interaction between the two factors (*F*_1,33_ = 0.004, *p* = 0.98) were found.

**FIGURE 2:**
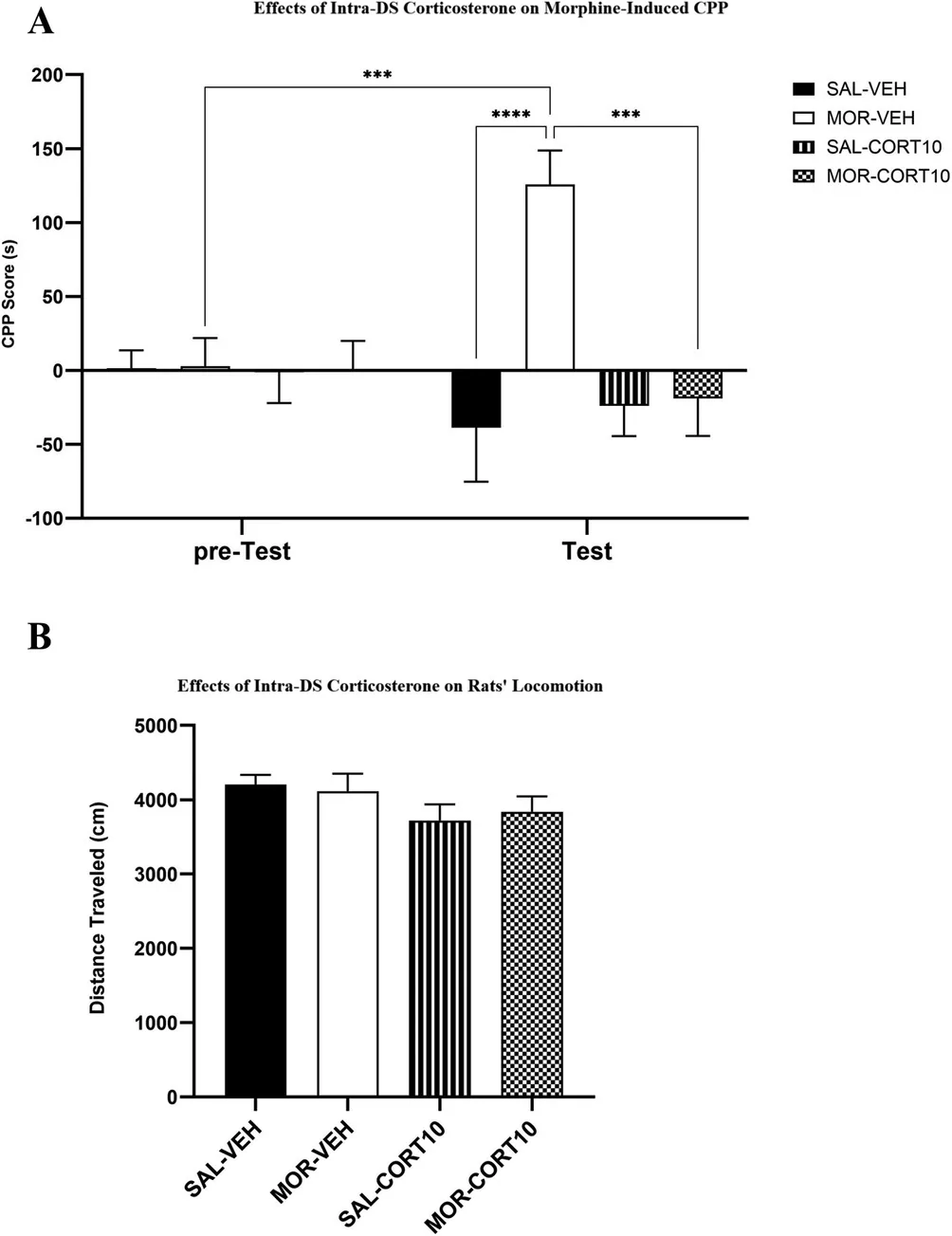
Impact of corticosterone injection into the dorsal striatum on the expression of morphine‐induced CPP. (A) CPP score during the pre‐test and test. (B) Rats' travelled distance during the post‐conditioning test. Bars represent mean ± SEM time spent in the drug‐paired chamber at pre‐test (Day 2) and Test (*n* = 9–10). ***p < 0.001 and ****p < 0.0001. CORT, corticosterone; CPP, conditioned place preference; intra‐DS, intra‐dorsal striatum; MOR, morphine; SAL, saline; VEH, vehicle.

A two‐way repeated measure ANOVA including two factors [Group (four groups) × Test (pre‐test and test)] conducted on the CPP score revealed a significant main effect of the Group (*F*_3,33_ = 4.56, *p* = 0.008) and a significant interaction between the Group and Test (*F*_3,33_ = 6.81, *p* = 0.001) (Figure 2A). Tukey's post hoc test demonstrated that the CPP score was significantly increased in the Test: MOR‐VEH group compared to the pre‐Test: MOR‐VEH group (*p* < 0.001) and the Test: SAL‐VEH group (*p* < 0.0001). Furthermore, the CPP score was significantly decreased in the Test: MOR‐CORT10 group compared to the Test: MOR‐VEH group (*p* < 0.001) (Figure 2A).

A one‐way ANOVA on the rats' travelled distance (locomotor activity) during the test showed no significant differences (*F*_3,33_ = 1.25, *p* = 0.30), indicating that the observed CPP effects cannot be attributed to alterations in general activity levels (Figure 2B).

### 3.2 Effects of Intra‐DS Injection of Spironolactone (MR Blocker) on the Expression of Morphine‐Induced CPP

Pre‐test analysis confirmed that baseline side preferences did not differ between groups. Second pre‐test, Day 2: no significant main effects of morphine (*F*_1,50_ = 0.01, *p* = 0.89) or spironolactone (*F*_2,50_ = 0.004, *p* = 0.99) or interaction between the two factors (*F*_2,50_ = 0.003, *p* = 0.99) were found.

**FIGURE 3:**
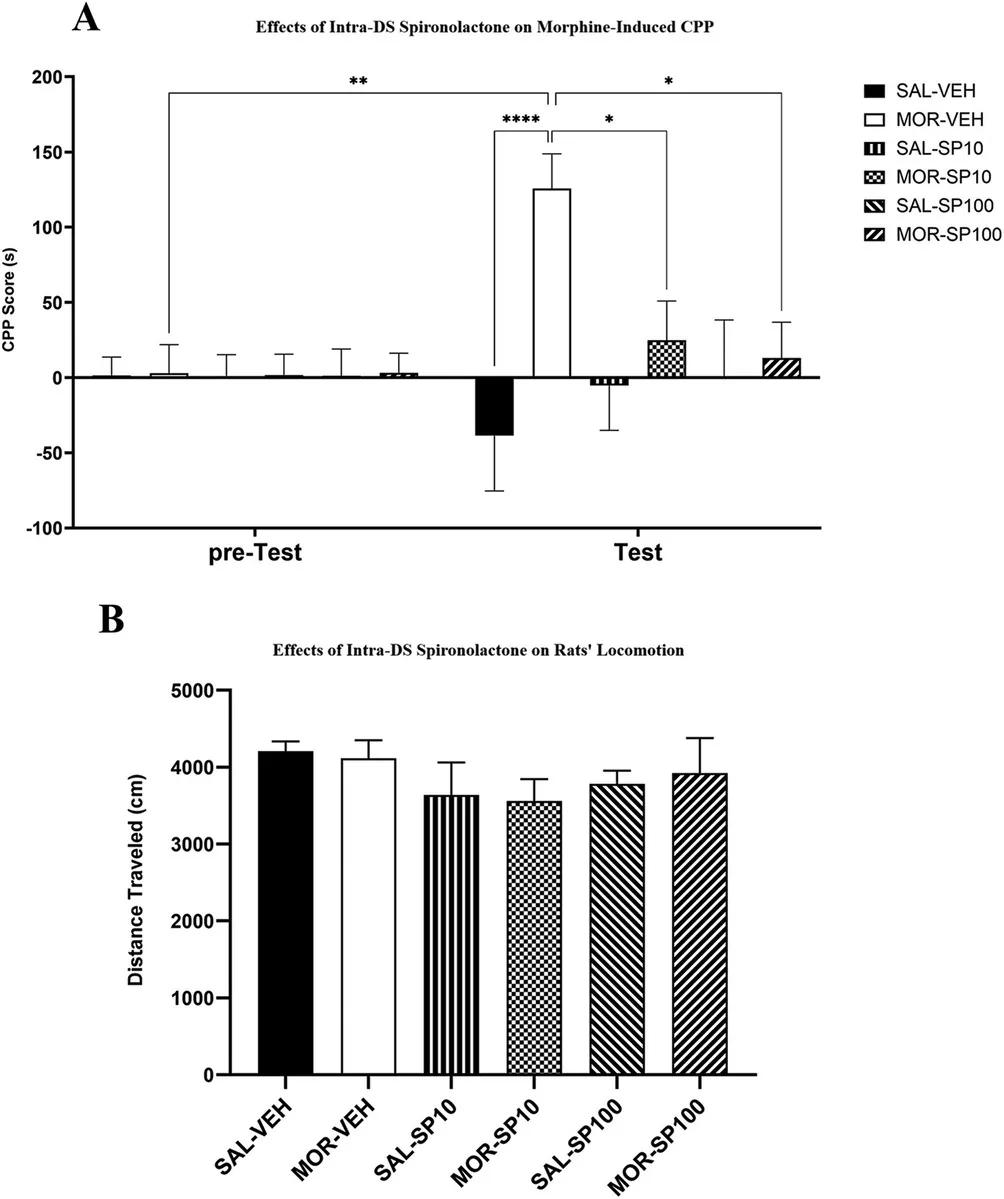
Impact of the spironolactone (MR blocker) injection into the dorsal striatum on the expression of morphine‐induced CPP. (A) CPP score during the pre‐Test and test. (B) Rats' travelled distance during the post‐conditioning test. Bars represent mean ± SEM time spent in the drug‐paired chamber at pre‐test (Day 2) and test (*n* = 9–10). ***p < 0.001 and ****p < 0.0001. CPP, conditioned place preference; intra‐DS, intra‐dorsal striatum; MOR, morphine; MR, mineralocorticoid receptor; SAL, saline; SP, spironolactone; VEH, vehicle.

A two‐way repeated measure ANOVA including two factors [Group (six groups) × Test (pre‐test and test)] conducted on the CPP score revealed a significant main effect of the Group (*F*_5,49_ = 2.89, *p* = 0.02) and a significant interaction between the Group and Test (*F*_5,49_ = 2.41, *p* = 0.04). Tukey's post hoc test demonstrated that the CPP score was significantly increased in the Test: MOR‐VEH group compared to the pre‐Test: MOR‐VEH group (*p* < 0.01) and the Test: SAL‐VEH group (*p* < 0.0001). Furthermore, the CPP score was significantly decreased in the Test: MOR‐SP10 and Test: MOR‐SP100 groups compared to the Test: MOR‐VEH group (*p* < 0.05) (Figure 3A).

A one‐way ANOVA on the rats' travelled distance (locomotor activity) during the test showed no significant differences (*F*_5,49_ = 0.76, *p* = 0.58), indicating that the observed CPP effects cannot be attributed to alterations in general activity levels (Figure 3B).

### 3.3 Effects of Intra‐DS Injection of RU38486 (GR Blocker) on the Expression of Morphine‐Induced CPP

Pre‐test analysis confirmed that baseline side preferences did not differ between groups. Second pre‐test, Day 2: no significant main effects of morphine (*F*_1,49_ = 0.004, *p* = 0.99) or RU38486 (*F*_2,49_ = 0.01, *p* = 0.98) or interaction between the two factors (*F*_2,49_ = 0.02, *p* = 0.98) were found.

**FIGURE 4:**
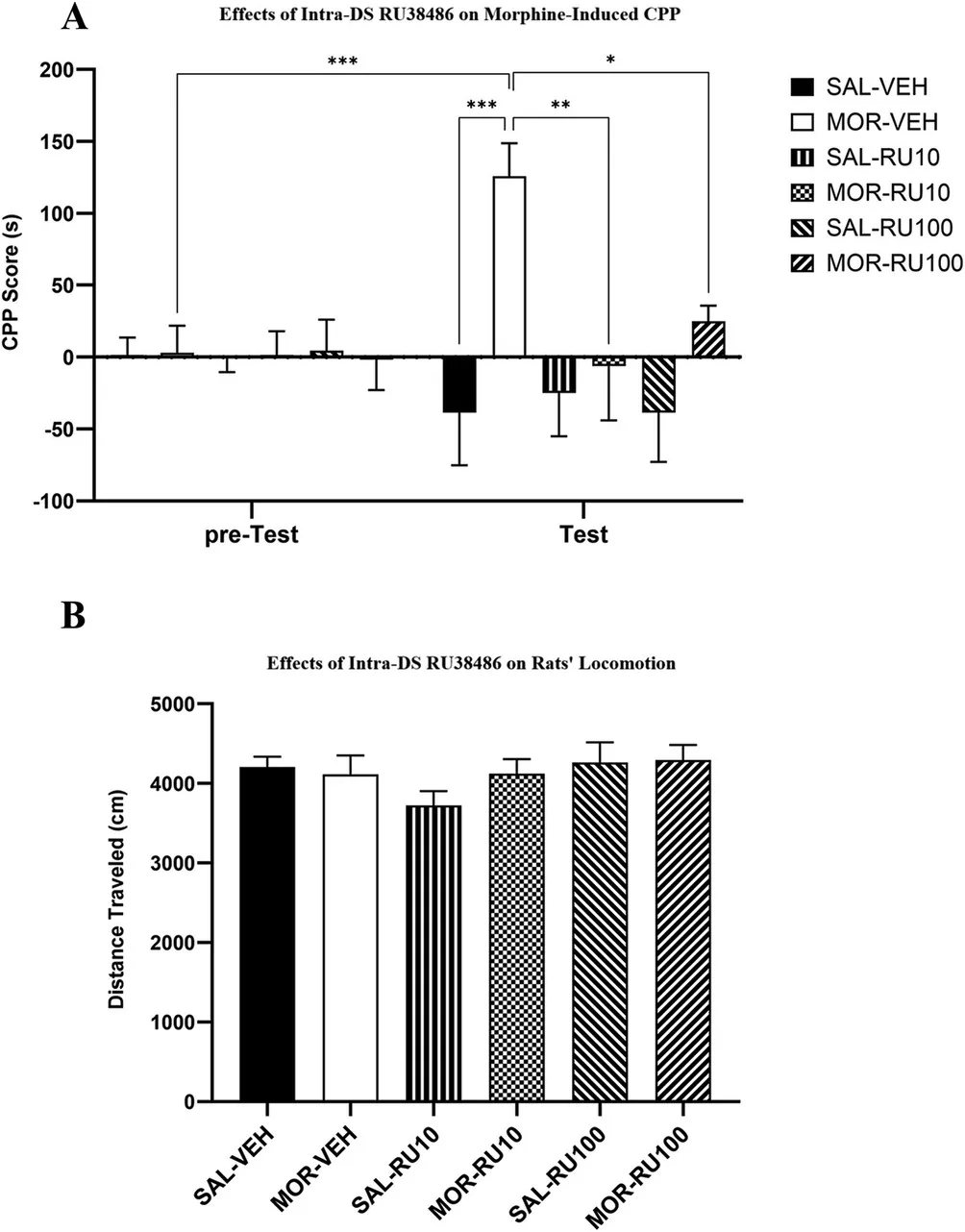
Impact of the RU38486 (GR blocker) injection into the dorsal striatum on the expression of morphine‐induced CPP. (A) CPP score during the pre‐test and test. (B) Rats' travelled distance during the post‐conditioning test. Bars represent mean ± SEM time spent in the drug‐paired chamber at pre‐test (Day 2) and test (*n* = 9–10). *p < 0.05, **p < 0.01 and ****p < 0.0001. CPP, conditioned place preference; GR, glucocorticoid receptor; intra‐DS, intra‐dorsal striatum; MOR, morphine; RU, RU38486; SAL, saline; VEH, vehicle.

A two‐way repeated measure ANOVA including two factors [Group (six groups) × Test (pre‐test and test)] conducted on the CPP score revealed a significant main effect of the Group (*F*_5,48_ = 3.1, *p* = 0.01) and a significant interaction between the Group and Test (*F*_5,48_ = 3.33, *p* = 0.01). Tukey's post hoc test demonstrated that the CPP score was significantly increased in the Test: MOR‐VEH group compared to the pre‐Test: MOR‐VEH group (*p* < 0.001) and the Test: SAL‐VEH group (*p* < 0.001). Furthermore, the CPP score was significantly decreased in the Test: MOR‐RU10 (*p* < 0.01) and Test: MOR‐RU100 (*p* < 0.05) groups compared to the Test: MOR‐VEH group (Figure 4A).

A one‐way ANOVA on the rats' travelled distance (locomotor activity) during the Test showed no significant differences (*F*_5,48_ = 1.36, *p* = 0.25), indicating that the observed CPP effects cannot be attributed to alterations in general activity levels (Figure 4B).

### 3.4 DAT Immunoreactivity in the DS

**FIGURE 5:**
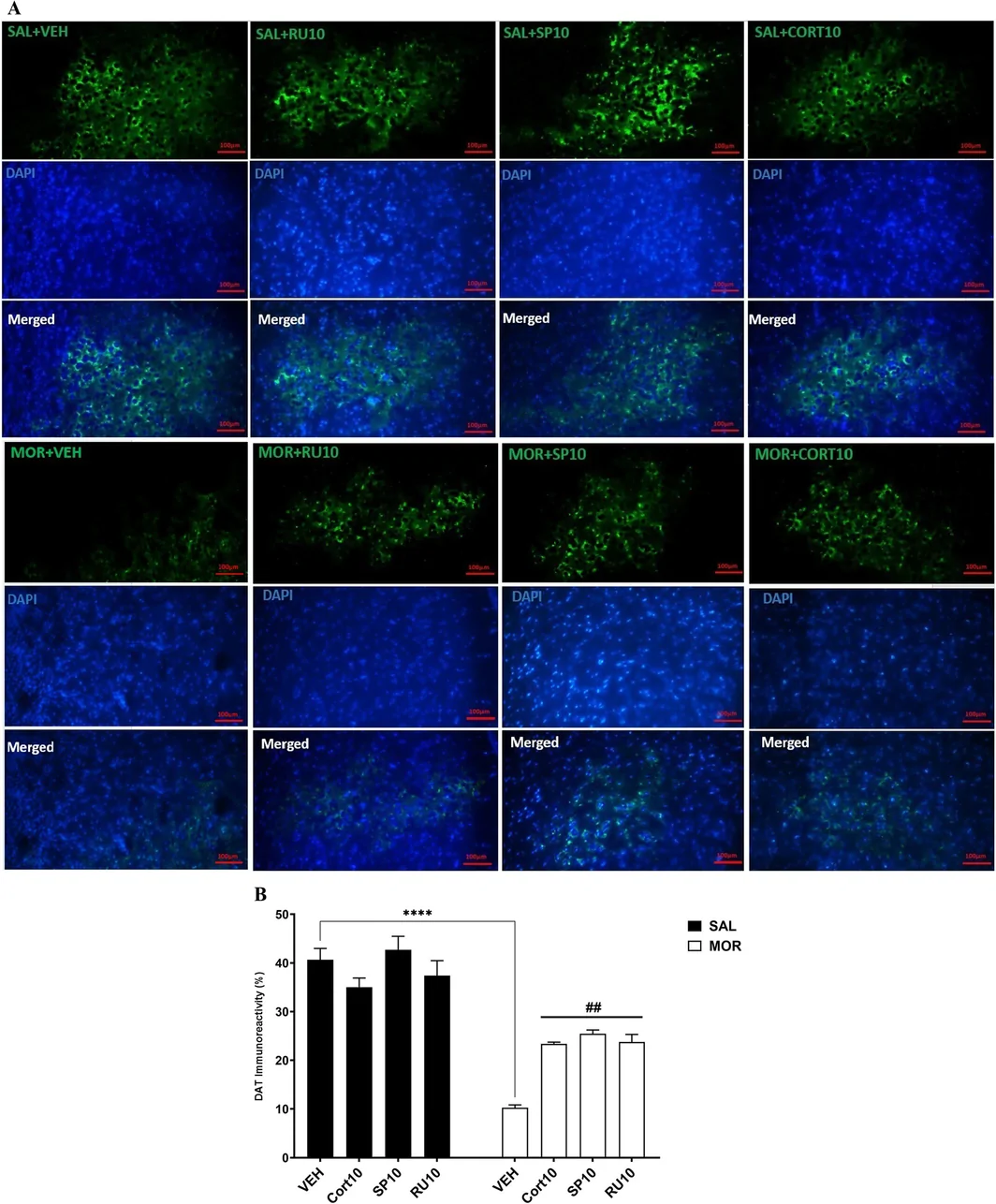
Representative DAT immunohistochemistry in the dorsal striatum. Images were acquired at 200× magnification under standardized exposure parameters. Scale bar = 100 μm. Brain tissues were collected from the same animals following completion of the conditioned place preference (CPP) procedure. (A) Representative images showing DAT immunostaining, DAPI nuclear staining and merged panels in the dorsal striatum. DAPI staining was used to confirm tissue integrity and anatomical localization, and merged images are presented for qualitative visualization. (B) Quantification of DAT immunoreactivity across experimental groups. Quantitative analysis was performed using the DAT fluorescence channel alone and conducted blind to group allocation. Data are expressed as the mean ± SEM (*n* = 4). ****p < 0.0001; ##*p* < 0.01 versus the MOR‐VEH group. CORT, corticosterone; DAT, dopamine transporter; MOR, morphine; RU, RU38486; SAL, saline; SP, spironolactone; VEH, vehicle.

Representative images of DAT immunostaining, DAPI nuclear staining and merged panels in the DS are shown in Figure 5A. DAPI staining was used to confirm tissue integrity and anatomical localization. In contrast, merged images are presented for qualitative visualization of the spatial relationship between DAT immunoreactivity and cellular structure. Quantitative analysis was performed using only the DAT fluorescence channel.

A two‐way ANOVA with factors Morphine (Saline vs. Morphine) and Corticosteroid manipulation (Vehicle, Corticosterone, RU‐38486, Spironolactone) revealed significant main effects of Morphine (*F*_1,24_ = 183.3, *p* < 0.0001) and Corticosteroid manipulation (*F*_3,24_ = 7.01, *p* = 0.001) and a significant interaction between the two factors (*F*_3,24_ = 9.80, *p* = 0.0002). Tukey's post hoc test demonstrated that the DAT immunoreactivity was significantly decreased in the MOR‐VEH group compared to the SAL‐VEH group (*p* < 0.0001). Moreover, the DAT immunoreactivity was significantly enhanced in the MOR‐CORT10, MOR‐SP10 and MOR‐RU10 groups compared to the MOR‐VEH group (*p* < 0.01) (Figure 5B).

## 4 Discussion

The central findings of this study are that (1) morphine induced CPP and reduced DAT immunoreactivity in the DS; (2) intra‐striatal corticosterone, spironolactone and RU‐38486 each attenuated morphine‐induced CPP; (3) these treatments also prevented the morphine‐induced decrease in DAT labelling; and (4) these effects occurred without locomotor confounds. Together, these findings indicate that perturbation of corticosteroid signalling within the DS can influence opioid reward processing and associated DAT plasticity. Importantly, however, the present pharmacological design does not establish whether the inhibitory effect of corticosterone is mediated by classical genomic GR signalling, rapid GR‐associated signalling or a GR‐independent mechanism. The convergence of corticosterone and corticosteroid receptor antagonists on similar behavioural and neurochemical outcomes should therefore not be interpreted as evidence that these treatments act through an identical receptor mechanism.

Morphine‐induced CPP is a well‐established behavioural measure of drug reward [27]. Our findings confirm this effect and extend prior work by showing that pharmacological manipulation of corticosteroid signalling within the DS alters morphine reward. Specifically, intra‐striatal corticosterone attenuated CPP (Figure 2A,B), consistent with reports that the DS plays a pivotal role in opioid reward and addictive behaviours [38]. These findings align with evidence that corticosteroid‐sensitive signalling can shape dopaminergic neurotransmission and reward‐related behaviour [21, 25, 26].

This interpretation is supported by earlier hypotheses that glucocorticoids act as modulators of reward processing. Piazza and Le Moal proposed that glucocorticoids are released in response to rewarding stimuli and influence dopaminergic activity, thereby facilitating adaptation to stress but potentially predisposing to addiction under chronic or excessive activation [39, 40]. Similarly, Marinelli et al. showed that GR blockade reduces dopamine release and morphine‐induced locomotor activity, highlighting an important contribution of GR signalling to opioid‐related dopaminergic responses [41]. Although these studies focused primarily on ventral striatal and mesolimbic circuits, our data indicate that corticosteroid‐sensitive mechanisms within the DS can also shape opioid reward. Nevertheless, because corticosterone activates both MR‐ and GR‐associated signalling and can exert rapid actions that are not readily explained by classical nuclear GR transcription, the present findings do not permit the corticosterone effect to be assigned specifically to GR activation.

Stress and opioid systems interact across multiple brain regions, including the amygdala, prefrontal cortex, striatum and nucleus accumbens [39, 42, 43]. Converging evidence demonstrates that stress and corticosterone exposure modulate opioid reward sensitivity through overlapping intracellular pathways. For example, Haghparast et al. demonstrated that acute and subchronic stress enhanced morphine‐induced phosphorylation of ERK and CREB and increased c‐Fos expression in mesocorticolimbic regions. However, subchronic stress paradoxically reduced CPP scores, suggesting that stress‐driven ERK/CREB/c‐Fos signalling differentially shapes molecular plasticity and reward behaviour [44]. Similarly, Li et al. reported that chronic, but not acute, footshock stress or corticosterone treatment potentiated morphine CPP via elevated dopamine release in the nucleus accumbens, implicating corticosterone‐driven dopaminergic activation as a mechanism linking stress and enhanced drug reward [45]. In contrast, Razavi et al. found that corticosterone in the basolateral amygdala inhibited morphine CPP, accompanied by altered c‐Fos and p‐CREB/CREB signalling [39].

Other interventions highlight the importance of mesolimbic dopamine modulation. Ma et al. demonstrated that peripheral electrical stimulation suppressed morphine CPP while normalizing dopamine and metabolite levels in the nucleus accumbens, emphasizing the central role of dopamine in both potentiating and suppressing morphine reward depending on the nature of the regulatory input [46]. Comprehensive reviews further support the bidirectional influence of stress. Bali et al. described how endogenous opioids such as β‐endorphins, enkephalins and nociceptin buffer hypothalamic–pituitary–adrenal (HPA) axis overactivation, whereas dynorphins released under chronic stress drive dysphoria and vulnerability to addiction. Importantly, acute stress generally inhibits morphine CPP, whereas chronic stress can enhance it through mechanisms involving GR signalling in the basolateral amygdala, ERK/CREB activation in mesocorticolimbic circuits and shifts in dopamine and GABAergic transmission [43].

Additional studies confirm that the effects of stress on morphine CPP depend on duration, controllability, and developmental history. Chronic or subchronic stressors, such as forced swim or restraint, often suppress CPP and engage ERK/CREB/c‐fos pathways [44, 47], whereas chronic footshock potentiates CPP via nucleus accumbens dopamine release [45]. Controllability is also critical: inescapable stress enhances morphine CPP, whereas escapable stress prevents it by regulating GABAergic transmission in the medial prefrontal cortex, a phenomenon termed ‘behavioural immunization’ [48]. Early‐life stress produces further variability, with maternal separation enhancing morphine CPP in some studies and attenuating it in others, supporting the ‘stress inoculation hypothesis’ [43].

Taken together, these findings underscore that stress‐opioid interactions are highly context dependent and are shaped by stress intensity, chronicity, controllability, brain region engagement and developmental timing. Our results extend this body of work by demonstrating that local pharmacological manipulation of corticosteroid signalling within the DS is sufficient to alter morphine CPP, thereby identifying the DS as an additional site of corticosteroid‐opioid interaction. The divergent findings reported across brain regions and experimental conditions also caution against a simple linear model in which increasing glucocorticoid signalling necessarily enhances reward and receptor blockade necessarily produces the opposite behavioural effect.

The present study demonstrates that pharmacological antagonism of either GRs or MRs within the DS reduced morphine CPP (Figures 3 and 4). Prior work has primarily emphasized GR‐mediated regulation of dopamine release and reuptake [26, 49]. Our findings suggest that MR‐sensitive signalling may also contribute to opioid reward, expanding the focus beyond GRs. This observation is consistent with evidence that both receptor types are expressed in corticosteroid‐sensitive neural circuits and that the relative balance between MR‐ and GR‐mediated signalling influences motivational and cognitive processes [24, 50].

The concept of corticosteroid receptor balance may be particularly relevant to the present findings. De Kloet et al. proposed that optimal neural adaptation depends not only on the absolute activation of one corticosteroid receptor subtype but also on a coordinated balance between MR‐ and GR‐mediated actions [13]. Because MRs generally exhibit higher affinity for endogenous corticosteroids than GRs, changes in corticosterone availability can progressively alter the relative recruitment of these receptor systems. Disturbance at either end of this functional range may therefore disrupt optimal neural processing. Within this framework, increased corticosteroid exposure and pharmacological receptor blockade need not necessarily produce mirror‐image behavioural effects; instead, both may displace corticosteroid signalling from an optimal functional range and converge on a similar behavioural endpoint. This interpretation remains hypothetical in the context of the present experiment but provides a biologically plausible framework for the observed findings.

As shown in Figure 5A,B, morphine conditioning decreased DAT immunoreactivity in the DS, consistent with prior reports that drugs of abuse alter DAT expression and function [51, 52, 53]. Notably, intra‐striatal corticosterone, RU‐38486 and spironolactone each attenuated this reduction. Because immunohistochemistry reflects relative optical density rather than absolute protein quantification, these results should be interpreted as preservation of DAT labelling rather than direct upregulation. Nevertheless, the convergence of CPP and DAT findings suggests that manipulation of corticosteroid‐sensitive signalling may stabilize dopamine regulation during opioid exposure, potentially constraining reward‐driven behaviours.

Previous studies have consistently shown that exposure to drugs of abuse, including opioids, alters DAT function and expression. Morphine and heroin reduce DAT levels and impair transporter‐mediated reuptake in striatal regions, contributing to sustained dopaminergic signalling and reinforcing drug‐associated cues [54]. Similarly, stress hormones have been implicated in modulating DAT expression: Corticosterone has been shown to alter DAT regulation in corticosteroid‐sensitive brain regions, thereby influencing extracellular dopamine availability [21, 22, 23]. The present results extend these findings by demonstrating that corticosteroid manipulations within the DS, a region critical for stimulus–response learning and the transition from goal‐directed to habitual drug use, can preserve DAT immunoreactivity during morphine conditioning.

The behavioural and neurochemical findings converge to suggest a functional link. Reduced DAT labelling following morphine exposure may reflect adaptations that alter dopamine clearance and facilitate reward‐associated behavioural plasticity. By attenuating this reduction, corticosterone and corticosteroid receptor antagonists may stabilize dopaminergic transmission, thereby diminishing morphine's rewarding effects as expressed in the CPP paradigm. This interpretation is consistent with reports that intact DAT function constrains excessive dopaminergic drive and protects against drug‐induced sensitization [55, 56].

The mechanisms underlying the convergent effects of corticosterone and receptor antagonists on DAT labelling remain unclear. One possibility is that corticosteroid signalling interacts directly with dopamine regulatory pathways. GRs and MRs influence the transcriptional and post‐translational regulation of DAT [21, 26]. However, corticosteroids can also exert rapid effects on neuronal activity and neurotransmission through non‐genomic mechanisms. Such actions may involve membrane‐associated corticosteroid receptors, cytosolic GR‐associated signalling, kinase pathways or other transcription‐independent processes [57, 58, 59]. Accordingly, the effect of corticosterone observed in the present study cannot be attributed specifically to classical genomic GR activation. Alternatively, GR or MR antagonism may induce distinct compensatory changes in corticosteroid‐sensitive circuits that ultimately converge on dopamine regulation. Thus, similar DAT and CPP outcomes do not necessarily imply a common proximal receptor mechanism.

Overall, these data support the view that DAT integrity may represent an important neurochemical substrate linking stress‐related corticosteroid signalling and opioid reward. By attenuating the morphine‐associated reduction in dorsal striatal DAT immunoreactivity, the pharmacological manipulations used here may constrain dopaminergic adaptations associated with drug reward. However, the precise receptor and signalling mechanisms responsible for the corticosterone effect require further investigation.

A particularly intriguing finding is that corticosterone and corticosteroid receptor antagonists produced similar effects on morphine CPP and DAT immunoreactivity. At first sight, the comparable effects of corticosterone and RU‐38486 appear paradoxical because corticosterone activates corticosteroid‐sensitive pathways, whereas RU‐38486 is conventionally used as a GR antagonist. Importantly, however, pharmacological agonism and antagonism do not invariably generate opposite behavioural endpoints. Neural systems are non‐linear; receptor effects depend on dose, receptor occupancy, cellular context and signalling timescale, and distinct molecular perturbations may converge on a common network‐level response. Several mechanisms, not mutually exclusive, may therefore be considered (Table 1).

**TABLE 1:** Proposed mechanisms underlying the convergent effects of corticosterone (agonist) and corticosteroid receptor antagonists (RU‐38486, spironolactone) on morphine‐induced conditioned place preference (CPP) and dopamine transporter (DAT) immunoreactivity in the dorsal striatum. The listed mechanisms are based on established concepts from the literature on corticosteroid receptors and reward circuitry (references are provided). These explanations are interpretive and were not directly tested in the present study; however, they are provided to contextualize the paradoxical convergence observed in our results.

Mechanism	Description	Refs.
Inverted U‐shaped dose–response	Both hypoactivation (antagonists) and hyperactivation (supraphysiological corticosterone) of GRs/MRs can disrupt optimal dopaminergic regulation, leading to similar behavioural outcomes	[57, 60, 61]
Receptor saturation/desensitization	Excess corticosterone may saturate receptors, triggering compensatory downregulation that functionally resembles receptor blockade	[62]
Genomic versus non‐genomic signalling	Corticosterone may act rapidly through non‐genomic mechanisms, whereas antagonists act more slowly through genomic inhibition; both pathways ultimately reduce dopaminergic drive to morphine.	[57, 58]
Homeostatic network adaptations	Blockade of GRs or MRs can induce compensatory changes in stress‐responsive circuits (dopamine, glutamate), paralleling the net effect of corticosterone	[9, 26]

Inverted U‐shaped dose–response and MR/GR balance: Corticosteroid effects on cognition and reward often follow a non‐linear or inverted U‐shaped function, in which both insufficient and excessive corticosteroid signalling can disrupt optimal neural processing [57, 60, 61]. This framework is closely related to the corticosteroid receptor balance hypothesis, which emphasizes coordinated MR and GR activity rather than isolated activation of either receptor [13]. Thus, corticosterone administration and receptor antagonism may perturb different positions within the same non‐linear regulatory system and nevertheless converge on similar CPP and DAT outcomes. Because only one corticosterone dose was examined, the present study cannot determine the position of this dose on the proposed dose–response curve, and the inverted U‐shaped explanation should therefore be regarded as an interpretive framework rather than a mechanism demonstrated by our data.

Context‐dependent pharmacology of RU‐38486: Although RU‐38486 (mifepristone/RU486) is widely used as a GR antagonist, its pharmacological behaviour is more complex than that of a pharmacologically silent competitive antagonist. Schulz et al. demonstrated that RU486 can exhibit partial GR agonist activity and showed that this activity depends on the GR N‐terminal domain and corepressor interactions [63]. Similarly, Zhang et al. reported that the relative glucocorticoid agonist activity of mifepristone increases with cellular GR abundance, demonstrating that its agonist‐versus‐antagonist profile can be influenced by receptor concentration and cellular context [64]. These observations do not establish that RU‐38486 acted as a partial agonist in the DS in our experiment. Nevertheless, they caution against interpreting the RU‐38486 findings as the simple pharmacological opposite of corticosterone and provide a plausible additional context for the convergent behavioural outcomes.

Classical genomic versus rapid corticosteroid signalling: Corticosterone can act through classical intracellular MR/GR‐mediated transcription, but corticosteroids also produce rapid, non‐genomic effects on neuronal signalling and neurotransmitter function [57, 58, 59]. Rapid corticosteroid actions may involve membrane‐associated receptors or intracellular receptor–associated signalling mechanisms and can engage kinase and second‐messenger pathways without requiring the slower transcriptional responses traditionally associated with nuclear GR activation. Therefore, our present data do not establish whether the inhibitory effect of intra‐striatal corticosterone on morphine CPP was mediated by classical GR signalling, rapid GR‐associated signalling, MR‐dependent mechanisms or a GR‐independent action. This distinction is particularly relevant because CORT and RU‐38486 were tested in separate groups.

Convergence of distinct signalling pathways: Finally, antagonism of GRs or MRs may initiate compensatory changes in corticosteroid‐sensitive dopamine and glutamate networks [9, 26], whereas corticosterone may acutely alter neurotransmitter release and corticosteroid receptor balance. These distinct upstream events could converge on a common downstream dopaminergic state that reduces morphine‐associated reward learning and preserves DAT immunoreactivity. Thus, the similar behavioural phenotype observed following CORT, RU‐38486, and spironolactone should be viewed as evidence of functional convergence rather than proof of an identical molecular mechanism.

Collectively, these considerations provide a more nuanced interpretation of the apparently paradoxical findings. The present results demonstrate that different pharmacological perturbations of corticosteroid‐sensitive signalling within the DS can converge on similar behavioural and DAT outcomes. They do not, however, establish whether the effect of corticosterone is mediated by classical GR activation. A direct CORT + RU‐38486 co‐administration experiment would be particularly informative in this regard. Determining whether RU‐38486 reverses, attenuates or fails to alter the effect of corticosterone could help define the contribution of RU‐38486‐sensitive GR signalling to the observed phenotype. Future studies combining this pharmacological approach with receptor‐specific molecular or genetic strategies will be required to distinguish classical GR‐mediated, rapid GR‐associated, MR‐dependent and GR‐independent mechanisms.

The absence of locomotor effects is a significant finding (Figures 2B, 3B and 4B). It indicates that the observed changes in CPP were unlikely to result from generalized changes in mobility. Prior studies have similarly reported that GR modulation can alter drug reward without affecting locomotor activity [41, 65], thereby supporting the behavioural specificity of the dorsal striatal effects observed here. Nevertheless, the lack of locomotor changes does not by itself identify the receptor or intracellular mechanism responsible for the effects of corticosterone.

Several limitations should be acknowledged. First, DAT immunohistochemistry was conducted on a relatively small subset of animals (*n* = 4 per group), which may limit statistical power and generalizability. Second, analyses were performed on the DS as a whole, although its subregions, particularly the dorsomedial and dorsolateral striatum, have distinct functional roles; future studies incorporating subregional approaches would provide greater mechanistic insight. Third, corticosterone was tested at a single dose, whereas antagonists were evaluated at two doses. Given the non‐linear nature of corticosteroid signalling and the importance of MR/GR balance, comprehensive dose–response analyses are warranted. Fourth, pharmacological manipulations lack the receptor and pathway specificity of genetic, chemogenetic or receptor‐selective molecular approaches. Finally, only male rats were used, limiting the generalizability of the findings across sexes.

A further mechanistic limitation, directly relevant to the convergent effects of corticosterone and RU‐38486, is that these compounds were evaluated in separate experimental groups and were not co‐administered. Consequently, the present data cannot determine whether the effect of corticosterone on morphine CPP is mediated by classical GR signalling, rapid GR‐associated mechanisms, MR‐dependent signalling or a GR‐independent pathway. In particular, the absence of a CORT + RU‐38486 group prevents us from determining whether the corticosterone effect is sensitive to GR blockade. Co‐administration of CORT and RU‐38486 therefore represents an important experiment for future investigation and, together with receptor‐specific molecular approaches, could clarify the contribution of GR‐dependent and non‐GR mechanisms.

## 5 Conclusion

In conclusion, this study demonstrates that pharmacological manipulation of corticosteroid‐sensitive signalling within the DS modulates morphine reward and associated DAT plasticity. Corticosterone, RU‐38486 and spironolactone attenuated morphine‐induced CPP and preserved DAT immunoreactivity, revealing a notable convergence of pharmacologically distinct manipulations. Rather than demonstrating that the corticosterone effect is definitively mediated through GR, the present findings suggest that distinct perturbations of corticosteroid signalling may converge on common behavioural and dopaminergic outcomes. Non‐linear MR/GR interactions, the context‐dependent pharmacology of RU‐38486 and rapid corticosteroid signalling provide plausible frameworks for interpreting this convergence. Direct CORT + RU‐38486 co‐administration and receptor‐specific mechanistic studies will be necessary to determine whether the corticosterone effect depends on classical GR activation or involves rapid GR‐associated or GR‐independent pathways. These findings broaden our understanding of corticosteroid–dopamine interactions in opioid reward and identify the DS as an important site for further mechanistic investigation.

## Author Contributions

**Payman Raise‐Abdullahi:** methodology, formal analysis, investigation, writing – original draft, writing – review and editing, formal analysis. **Fatemeh Rezamohammadi:** methodology, investigation, formal analysis, writing – original draft. **Mehrnoush Rahmani:** methodology, investigation, formal analysis, writing – original draft. **Abbas Ali Vafaei:** methodology, investigation, writing – original draft. **Roghayeh Pakdel:** methodology, investigation, writing – original draft, investigation. **Hamed Rashidipour:** methodology, investigation, writing – original draft. **Ali Rashidy‐Pour:** conceptualization, formal analysis, writing – review and editing, writing – original draft, investigation, supervision.

## Funding

This study was supported by grants from the Semnan University of Medical Sciences, Semnan, Iran (grant number 1729).

## Ethics Statement

All experimental procedures were conducted in accordance with the National Research Council's *Guide for the Care and Use of Laboratory Animals* and were approved by the Research Ethics Committee of Semnan University of Medical Sciences (approval number: IR.SEMUMS.REC.1398.260). This study did not involve human participants, human data or human tissue.

## Conflicts of Interest

The authors declare no conflicts of interest.

## Data Availability

The datasets generated and/or analysed during the current study are available from the corresponding author on reasonable request.
